# TFClass: expanding the classification of human transcription factors to their mammalian orthologs

**DOI:** 10.1093/nar/gkx987

**Published:** 2017-10-26

**Authors:** Edgar Wingender, Torsten Schoeps, Martin Haubrock, Mathias Krull, Jürgen Dönitz

**Affiliations:** Institute of Bioinformatics, University Medical Center Göttingen, Georg August University, D-37077 Göttingen, Germany; geneXplain GmbH, D-38302 Wolfenbüttel, Germany; Dpt. of Evolutionary Developmental Genetics, Johann-Friedrich-Blumenbach Institute of Zoology and Anthropology, Georg August University, D-37077 Göttingen, Germany

## Abstract

TFClass is a resource that classifies eukaryotic transcription factors (TFs) according to their DNA-binding domains (DBDs), available online at http://tfclass.bioinf.med.uni-goettingen.de. The classification scheme of TFClass was originally derived for human TFs and is expanded here to the whole taxonomic class of mammalia. Combining information from different resources, checking manually the retrieved mammalian TFs sequences and applying extensive phylogenetic analyses, >39 000 TFs from up to 41 mammalian species were assigned to the Superclasses, Classes, Families and Subfamilies of TFClass. As a result, TFClass now provides the corresponding sequence collection in FASTA format, sequence logos and phylogenetic trees at different classification levels, predicted TF binding sites for human, mouse, dog and cow genomes as well as links to several external databases. In particular, all those TFs that are also documented in the TRANSFAC^®^ database (FACTOR table) have been linked and can be freely accessed. TRANSFAC^®^ FACTOR can also be queried through an own search interface.

## INTRODUCTION

Transcription factors (TFs) are proteins that regulate transcription, e.g. by directing RNA polymerase to the transcription start site of a gene. Most TFs do so by recognizing regulatory elements in promoters and enhancers in a sequence-specific way through their DNA-binding domains (DBDs). These DBDs are organized by few structural principles, which can be used to classify DNA-binding TFs as we have done with TFClass. Previously, we have described the underlying classification scheme and its application to human TFs (1) and to their rodent orthologs (2). Criteria for classifying TFs have been discussed elsewhere (3). In this report, we present the extension of TFClass to all mammals as far as annotated genomic information is available. The TFs assigned in TFClass are linked to entries in the FACTOR table of the TRANSFAC^®^ database, the oldest actively maintained resource for TFs and their DNA-binding sites and properties (4).

## METHODS

### Data sources

Using the catalog of human TFs from previous versions of TFClass as starting point, we retrieved the corresponding ortholog clusters from OrthoDB. v8 (5). The collected entries from up to 41 mammalian species were semi-manually pruned for any entries that were highly truncated or contained stretches of >5 undefined positions or any undefined positions within their DNA-binding domains (DBDs). If necessary, further paralog assignments were done manually after several iterative phylogenetic analyses.

### Domain annotation

As reported for the previous releases, domain assignments, protein sequences, and information about isoforms are taken from UniProt, last update done using release July 2014 (6), and from TRANSFAC^®^, with the last update using release 2017.2 (4). By searching for the orthologous domain boundaries in the alignments and subsequent extensive manual editing, the DBD sequences were compiled as FASTA files. For the visualization of the isoforms and for marking the DBD the entries are retrieved dynamically from UniProt as RDF file (Resource Description Framework). The downloaded file is cached and invalidated after 3 months to reflect updates in the database of UniProt in TFClass. For the marking of DBD the sequence of the compiled DBD FASTA file is mapped to the isoform sequences of UniProt. The Ensembl IDs, provided by OrthoDB were mapped to UniProt using BioMart in R and joined with the manually curated data of the previous version of TFClass. If the mapping resulted in more than one UniProt ID, the best one was selected based on the metadata of the UniProt entry.

### Phylogenetic analyses

Alignments of whole molecule and DBD sequences and subsequent phylogenetic analyses were mainly done with a local installation of the ‘one-click’ pipeline provided by Phylogeny.fr (7) and webPRANK tool at EBI (8). For their visualization we used the online tool iTOL (Interactive Tree Of Life) (9). After alignment of the DBDs, logo plots were generated with the aid of a locally installed and customized version of WebLogo (10).

### TFBS prediction

As reported before (2), we used the TRANSFAC^®^ matrix library (release 2012.2) with a reprogrammed version of the Match algorithm (11) to predict potential TF binding sites (TFBSs) for 1359 vertebrate matrices using the minFN profile. The predicted conserved TFBSs in the human (hg19), mouse (mm9), dog (canFam2) and cow (bosTau4) genome for each TF were also assigned to the other members of the corresponding TF (sub-)family in TFClass, since by definition they have highly similar, or even identical, DBDs (‘paralog expansion’, see (12)).

## STRUCTURE OF THE CLASSIFICATION

Generally, the structure of the classification has been kept as in the previous releases. The classification tree represents a five-level hierarchy of Superclasses, Classes, Families, (optional) Subfamilies and Genera. The nine structurally defined superclasses are still complemented by a tenth superclass labelled ‘0. Yet undefined DNA-binding domains’, where TFs have been compiled known to bind to DNA, but the structure of their DBD has not yet been investigated or, in case of one class, not even identified. The contents of this superclass will be moved as soon as this knowledge is available. The individual orthologous mammalian TF genes are linked to the Genus level. A sixth level, that was consistently populated with human TFs and was called (molecular) Species, represents the different proteins encoded by a TF gene due to alternative splicing. Since these isoforms largely differ between the biological species covered now by TFClass, this level has been resolved into a comparative visualization of the splice variants with reference to the position of the respective DNA-binding domain, dynamically retrieved from UniProt. This illustrates whether certain splicing events may affect DNA-binding of the corresponding TF.

## ONTOLOGY BACKEND

TFClass has been put into an ontological framework, modeling the structure of the classification and also containing its meta data (e.g. class definitions or consensus sequences), the species-specific factors including links to other information resources, and some information about the biological species taken from the NCBI Taxonomy (https://www.ncbi.nlm.nih.gov/taxonomy). The ontology is loaded by the OBA (Ontology-Based Answers) server (13), which is used to dynamically serve the classification, searches therein and computations like the number of species for which a TF is documented in TFClass.

## MAMMALIAN ORTHOLOGS OF HUMAN TF

After refinement of the groups of orthologs from OrthoDB (see Methods), we came up with TFs for species from 18 out of the up to 30 presently discussed mammalian orders. Of them, the primates are best covered (10 species), followed by rodents and Artiodactyla/even-toed ungulates (five species each). On average, we have classified about 950 TFs from each species (Table 1).

**Table 1.** Mammalian orders and the number of assigned TFs

Order	Biological species	TFs
Afrosoricida	1	440
Artiodactyla	5	4780
Carnivora	4	4710
Chiroptera	2	1819
Cingulata	1	1321
Dasyuromorphia	1	990
Didelphimorphia	1	1223
Diprotodontia	1	405
Eulipotyphla	2	634
Hyracoidea	1	529
Lagomorpha	2	1631
Monotremata	1	823
Perissodactyla	1	1215
Pilosa	1	288
Primates	10	11765
Proboscidea	1	1103
Rodentia	5	5031
Scandentia	1	330
**Sum**	**41**	**39037**

Listed are those mammalian orders to which the 41 biological species covered here belong to. Also given are the number of species of each order and the number of TFs assigned to these species.

The groups of orthologs that were retrieved from OrthoDB for each human TF were subjected to a phylogenetic analysis at subfamily, family and class level, not for superclasses since they are mainly defined according to their structural similarities. The resulting phylogenetic trees are provided for each node of the classification, for both the complete protein sequences as well as for the DBDs only. The individual subtaxa are given standardized background colors. In cases that all or some TFs in one cluster contain more than one DBD, these may be listed in the FASTA file as distinct entities and are distinguished in the phylogenetic trees with distinct label colors. Each class as well as many families comprise too many individual TFs to be displayed in one tree. In these cases, a ‘slim selection’ was compiled consisting of the human, mouse, cow and *Monodelphis* sequences only. If one of them was not available, it may have been substituted by a related species. Altogether, we have incorporated into TFClass the visualized results of 1192 phylogenetic analyses, 568 of them for the full-length proteins, 624 for their DBDs. The results obtained with several methods are shown in cases where there appeared ambiguities, otherwise only one phylogenetic tree is displayed. 780 FASTA files (440 full-length proteins, 340 DBDs) are linked to the respective node in TFClass.

From each set of aligned DBD sequences, we generated a sequence logo to indicate the most conserved residues in each group. For generating logos for subfamilies, individual sequences with insertions were removed from the alignment in order to retain the consensus. At higher levels (Family or Class), it may happen that several of the sequences, for instance those of one subfamily, consistently have inserts at a certain position of the consensus, which have been kept when generating these higher-level sequence logos. Altogether, TFClass presently provides 339 sequence logos.

On top of the page for each node, the number of biological species contributing TFs to this group is given. In larger classes or families, this number usually approaches the maximum of 41. At the genus level, the provided information about the number of biological species indicates whether a certain human TF has orthologs in all mammals, which is the case for most TFs, or whether some TFs have been emerged late during evolution. For instance, most TFs have ∼30 species within one Genus. The TFs of the zinc finger subfamily 2.3.3.27, however, have known orthologs in only six biological species, all of them primates.

A few caveats should be made here: (i) In particular among the TFs with numerous contiguous zinc finger modules, the clusters of apparent orthologs retrieved from OrthoDB are sometimes huge and subsume homologs of many similar, but distinct human TFs. Further efforts are still required to classify them. (ii) Since the starting point for the retrieved clusters of orthologs were human TFs, TFClass may lack TFs that are specific for other orders than primates. This may especially pertain to the zinc finger class 2.3. (iii) Because of the ongoing research on proper clustering of zinc finger TFs, the families and subfamilies in Class 2.3 are not yet completely covered by the phylogenetic analyses documented in TFClass. (iv) Visual inspection of the phylogenetic trees in TFClass for all available mammalian sequences confirms the families and subfamilies previously defined for human TFs in the majority of cases. However, a few instances may require future re-assignment in the light of their evolutionary history.

## TRANSFAC

The TRANSFAC^®^ database is the oldest still actively maintained collection of information about eukaryotic TFs and their DNA binding sites, also including the largest library of DNA binding profiles (positional weight matrices) available so far (4). With this new release of TFClass, we also make publicly accessible the TF information of the FACTOR table of TRANSFAC^®^. The fields published correspond to those of the public domain FACTOR table (from 2007, http://www.gene-regulation.de), but the contents are from the latest state of the proprietary TRANSFAC version (release 2017.2). In this release, the FACTOR table comprises 25,069 entries, among them 8800 human TF records. These entries are displayed in the TRANSFAC flat file format (http://genexplain.com/wp-content/uploads/2016/09/transfac_documentation_2012-03.pdf) and are either accessible through the TRANSFAC links provided in TFClass or by an own search web interface (http://factor.genexplain.com).

## TF BINDING SITES

Because of the paralog expansion of the predicted TFBSs, the lists of potential binding sites are linked to the corresponding subfamily node or, if this has not been defined, to the family level. Altogether, 1349 PWMs of the TRANSFAC^®^ matrix library were used to come up with about 160 million predicted sites in the genomes of human, mouse, dog and cow, linked to 229 TFClass nodes, which can be filtered according to their ranked Match score.

## INTERFACE

**Figure 1.**
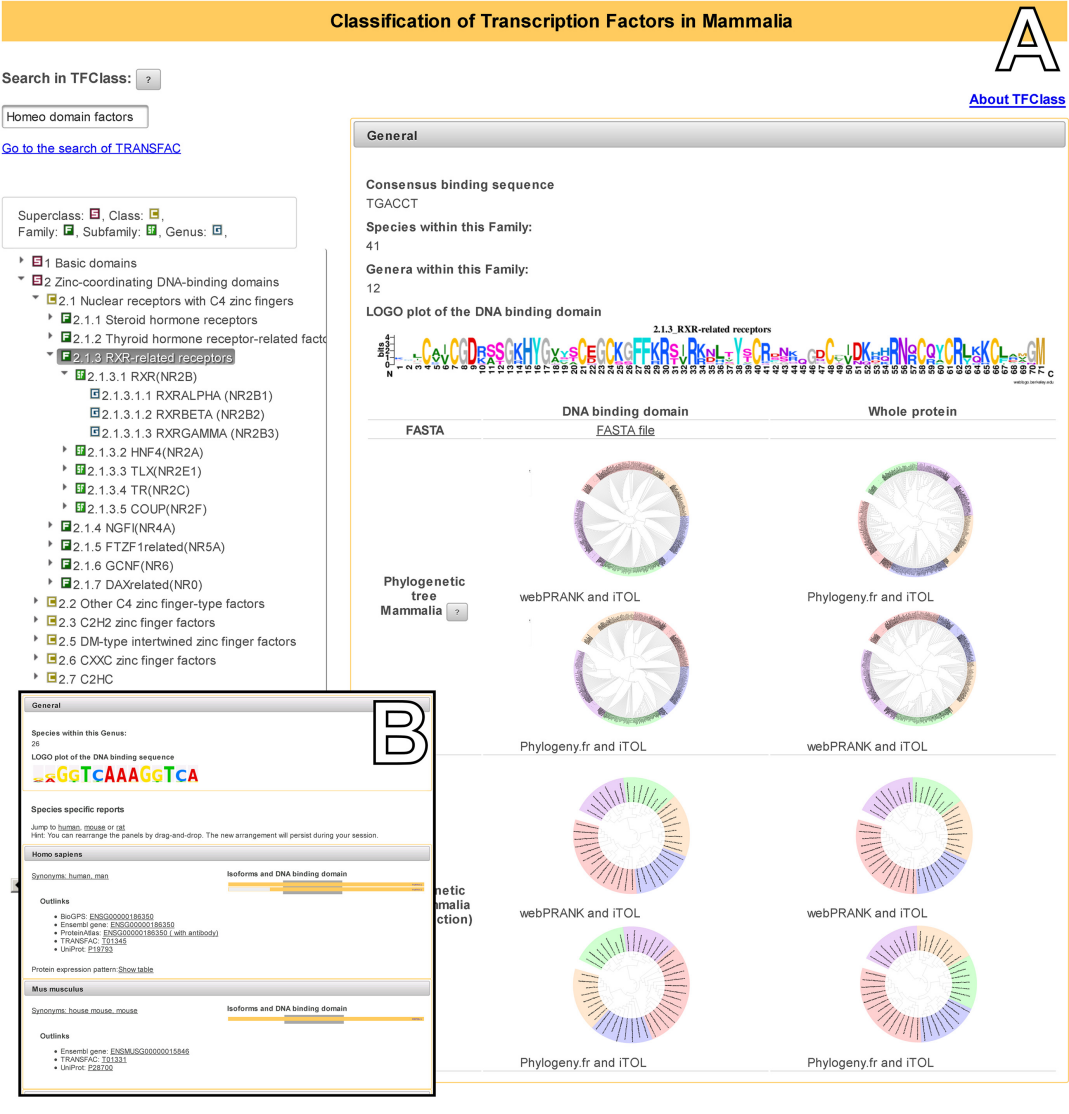
Screenshot of a Family page (**A**) and a Genus list (**B**) in TFClass. (A) The example shows the page for family 2.1.3, RXR-related nuclear receptors with some summarizing information in the upper and thumbnails of the phylogenetic trees in the lower part. Here, the upper four trees refer to all TFs of this family, whereas the lower four trees show the relations between the corresponding TFs from human, mouse, cow and *Monodelphis* only (‘slim selection’). (B) The insert shows the beginning of the list of species-specific instances for RXRalpha (2.1.3.1.1), with human and mouse entries put at the top of the list. The top yellow bar on the right of each entry represents the ‘canonical’ form of the corresponding UniProt entry. For the splice variants underneath, those parts of the molecule that diverge from the canonical form are highlighted by light-grey areas, the position of the DBD is indicated by the dark-grey box behind the bars.

When accessing TFClass, a session is established. Starting from a list of Superclasses, the classification tree can be expanded by clicking on the arrowhead in front of the individual items, or to get information about the item by clicking on its name. For classes, families and subfamilies, definitions, the number of biological species and TF genera under the corresponding node, sequence logos (as png files) and thumbnails of the phylogenetic trees (in svg format) are given (Figure 1). Enlarged views of graphics can be invoked and can be further manipulated by a zoom in/zoom out function. At the lowest level of the classification tree, the Genus level, a list of TFs assigned to the different biological species is given, the species name being linked to the NCBI taxonomy, and links to external databases are provided. The position of individual list entries can be changed by the user, for instance the human entry can be shifted to the top of the list. The new order is maintained throughout the session.

## AVAILABILITY

TFClass is freely accessible at http://tfclass.bioinf.med.uni-goettingen.de; single nodes of the classification can be addressed with the query parameter tfclass, e.g. http://tfclass.bioinf.med.uni-goettingen.de/?tfclass=1.2.3. The ontology has been made available in Turtle format as a downloadable file. TRANSFAC FACTOR entries are accessible through the links given in TFClass or directly under http://factor.genexplain.com.
